# Point-of-Care Ultrasound for Tropical Disease: Implications for Clinical Decision-Making

**DOI:** 10.4269/ajtmh.20-0303

**Published:** 2020-05-04

**Authors:** Daniel Kaminstein

**Affiliations:** Department of Emergency Medicine, Medical College of Georgia at Augusta University, Augusta, Georgia

Point-of-care ultrasound is increasingly being used by a spectrum of providers to help in clinical decision-making. In this issue of the *Journal*, a short report by Husan et al. outlines the ultrasound findings in 18 patients with culture-confirmed melioidosis who presented to a single hospital in Laos with fever during a 5-month period in 2016.^1^ The authors describe how findings of solid organ abscesses in patients presenting with fever had a high positive predictive value for diagnosing melioidosis. They conclude that providers in endemic regions should consider empiric antibiotic coverage for *Burkholderia pseudomallei* in febrile high-risk patients when a solid organ abscess is found. Other studies that have looked at imaging in patients with melioidosis support these recommendations.^2–4^ Those new to ultrasound use by clinicians may be skeptical as to why a study that incorporates only 18 patients should prompt a specific treatment recommendation. In the following paragraphs, I will outline some specific considerations when applying these results, with a focus on current and future implications.

We live in uncertain times. As I write this commentary, we are in the midst of a global pandemic. We have seen a virus identified in China become the greatest global health challenge of our time. So why should we read about ultrasound for a relatively uncommon bacterial condition seen mostly in parts of Asia and Australia? In fact, this article could not be better timed. Western medicine has long relied on our abundant resources as a primary way to advance medical care. For the first time in a long time, high-resource countries are struggling with the realities of decision-making and clinical care when resources are severely limited. Simple technologies such as point-of-care ultrasound have proven to be invaluable in such scenarios.^5–9^

There is a growing body of evidence describing the use of ultrasound by clinicians for the diagnosis and treatment of tropical infectious diseases in both adult and pediatric populations.^10–12^ Applications of ultrasound for tropical diseases include evaluations of almost every major organ system including the heart, lungs, liver, spleen, kidneys, and even the skin. The earliest use of ultrasound for tropical disease focused on ultrasound findings in the liver.^13–15^ The described sonographic changes of periportal fibrosis seen in *Schistisoma mansoni* infections and characteristic cystic structures seen in *Echinococcus granulosus* infections remain important for both diagnosis and treatment.^14,16^ More recently, in tropical and resource-limited settings, the focus has shifted away from ultrasound as a pure diagnostic tool toward its utility in helping providers with clinical decision-making.

With improved machine portability and image quality, indications for ultrasound and use by healthcare providers have both grown substantially. In 2010, Heller et al.^17,18^ initially described the Focused Assessment with Sonography for HIV-associated TB (FASH) examination for extrapulmonary tuberculosis. This was during a massive outbreak of HIV infection and tuberculosis in South Africa. Resources were limited, patient presentations were nonspecific, and treatment decisions had to be made based on limited information. The FASH examination was used for examining the heart, lungs, and abdominal organs for signs of extrapulmonary tuberculosis. If found, and when other diagnostic tests were unavailable or unrevealing, providers would start empiric antituberculous treatment. The initial description of the FASH examination included an evaluation of liver lesions.^17^ Later versions were modified to focus more on high-yield findings, and a linear probe was used to more accurately identify splenic lesions.^10^ Follow-up studies showed that serial examinations might be helpful in monitoring treatment effects.^19^

The value of research such as that described by Husan et al.^1^ in this issue includes the establishment of a foundation on which future research can be based. At the same time, it proposes a way in which providers can expand beyond the traditional history and physical examination to make better decisions based on an uncertain clinical scenario, with high stakes and limited resources. In this sense, it mirrors the approach that was the basis of the FASH examination, which is now accepted widely in South Africa as a valuable diagnostic tool.^20^ For a provider at the bedside, the specific diagnosis may be less important than knowing how to best make appropriate clinical decisions. A primary component of being a clinician is knowing how to use the resources you have to make the best decision for a patient in front of you. Small studies like this one give us a window into how simple tests, such as ultrasound, can contribute directly to patient care.

So, why is the article by Husan et al. on ultrasound appearing in a tropical medicine journal?^1^ As tropical medicine physicians, we are best positioned to understand the value of context in a patient’s presentation. We can ask why does this patient present with these symptoms in this place? Knowing how diseases differ in different parts of the world is what we do best.

The study by Husan et al. was undertaken in Southeast Asia, where melioidosis is relatively common.^1^ The differential diagnosis for a liver abscess seen by ultrasound is fairly extensive and includes diseases such as brucellosis, alveolar echinococcosis, fascioliasis, amebic abscess, bacterial abscess, and fungal infection, to name just a few. A similar study in Africa, with different patterns of disease, would not have yielded the same findings. In Africa, a patient presenting with fever and a liver abscess would more likely have either an amebic or bacterial abscess. If characteristic central calcification was seen, then brucellosis might be suspected. Around Lake Victoria, periportal fibrosis in the liver would be highly suspicious of a *Schistosoma mansoni* infection, and double-walled liver cysts in southern Italy would suggest *E. granulosus* infection. Thus, in a different geographical environment, the specific protocol mentioned in this article might lead to different conclusions. We should embrace this ambiguity rather than feel uncomfortable with it. This is what we are trained to do in tropical medicine. We use the distribution of a disease to help us make decisions.

Point-of-care ultrasound is not a tool that is designed to yield a definitive diagnosis. It is a tool that helps to refine a differential diagnosis based on the image and the context. Finding solid organ abscesses in the liver, spleen, or prostate of a diabetic patient with fever in Laos should prompt empiric treatment for *B. pseudomallei*. Ultrasound will not always be the answer, of course. Studies of other common diseases that cause fever in Southeast Asia such as dengue and malaria have not demonstrated clear utility of ultrasound in helping with either diagnosis or treatment.^21–25^

This article adds to our growing understanding about how point-of-care ultrasound should be used to guide treatment decisions in patients with undifferentiated fever, in particular in tropical settings with limited resources. Different locations and the diseases that occur in these locations may lead to similar patterns of ultrasound findings identifying different diseases. It is up to us as clinicians to put all of the information together, synthesize that information, and make the best treatment decisions.

More studies on the use of point-of-care ultrasound to aid in the diagnosis of tropical infections are needed, but pending these studies, the approach offered by Husan et al. is reasonable.^1^ A patient seen in an area with a high risk of melioidosis with a solid organ abscess on ultrasound should be treated presumptively for this diagnosis. More broadly, point-of-care ultrasound is becoming accessible to a growing body of clinicians with an expanding list of potential applications.
